# Influence of Temperature on the Damping Properties of Selected Viscoelastic Materials

**DOI:** 10.3390/ma17235832

**Published:** 2024-11-28

**Authors:** Lucjan Witek, Piotr Łabuński

**Affiliations:** 1 Department of Aerospace Engineering, Rzeszow University of Technology, al. Powst. Warszawy 8, 35-959 Rzeszów, Poland; 2 Doctoral School of Engineering and Technical Sciences, Rzeszow University of Technology, al. Powst. Warszawy 12, 35-959 Rzeszów, Poland; pj.labunski@gmail.com

**Keywords:** damping materials, butyl rubber, bituminous material, passive vibration isolation, influence of temperature, experimental modal test, free layer damping (FLD)

## Abstract

The paper presents results of experimental investigations of the influence of temperature on the effectiveness of passive vibration isolation. Two types of viscoelastic materials (butyl rubber and bituminous material) were tested. In the performed vibration analysis, the Oberst beam made out of aluminum alloy with a damping material in a Free Layer Damping (FLD) configuration was used. The experimental modal analysis was performed using the Unholtz-Dickie UDCO TA-250 vibration system. To investigate the influence of temperature on the effectiveness of passive vibration isolation, an isothermal cooling chamber (using Peltier cells) was designed and constructed. The tests were carried out in a wide frequency range from 40 Hz to 4000 Hz, at a constant sweep rate, in a temperature range from −2 °C to 22 °C. Miniature piezoelectric acceleration sensors were used to determine the acceleration of the beam and the exciter head. The analysis of accelerations of both the object and the shaker head allowed for the determination of a Frequency Response Function (FRF) for the beam. The course of FRF was used to determine the resonance frequencies and the vibration amplitudes of the beam damped with bituminous material and butyl rubber at various temperatures. The loss factor η, calculated for each resonance using the generalized half-power method (n-dB method), was used as an indicator of damping intensity. The research results presented in this work (important from scientific point of view) also have utilitarian significance and can be used in the design of more quiet and comfortable motor vehicles, railway wagons and aircraft structures.

## 1. Introduction

Vibrations may have various sources and be the result of natural phenomena or the operation of machine parts. They are transmitted in various ways, generating noise and directly affecting the human body and the structure of the vehicle. Long-term exposure to a noisy environment has a negative impact on well-being and physical and mental health. Increasing acoustic comfort and noise reduction is becoming increasingly important [1,2,3]. Noise in cars is usually related to engine operation or driving on uneven surfaces (transfer of vibrations from the suspension system to the body).

Vibration damping is particularly important in aviation. Long-term work of a pilot in a noisy and vibrating environment adversely affects his physical condition and increases fatigue. This reduces his ability to make the right decision and act appropriately when controlling the flight. Studies have confirmed that long-term exposure to vibrations may cause neck and spine injuries in the pilot or crew [4,5]. In order to ensure safety, it is necessary to determine the vibration level comfortable for humans [6]. There are two main sources emitting acoustic waves in an airplane [7,8,9]. One of them is mechanical vibrations of the structure, caused, for example, by rotating engine turbines. The second is turbulence, caused by, e.g., the flow of exhaust gasses or the rotation of a propeller.

Increasing the vibration amplitude causes stress pulsations and reduces the fatigue life of the structure. Resonant vibrations induced by an unbalanced aircraft engine rotor, in addition to noise in the cabin, also reduce the fatigue life of key engine parts, such as compressor blades [10].

The most common solution used to dissipate vibration energy is to cover the vibrating surface with a mat made of viscoelastic material. It is highly effective and inexpensive to apply. This type of vibration isolation is sensitive to changes in environmental conditions; therefore, during its implementation, it is necessary to take into account the influence of temperature on its effectiveness [11].

The most often polymers used in damping are butyl-based materials and bitumen [12]. From an economical point of view, viscoelastic materials are applied in over 85% of the passive damping of vibrations [13]. When designing or operating a structure in which passive vibration isolation is planned, knowledge about the damping properties of viscoelastic materials is needed. The loss factor η is often used as an indicator of structure damping and can be used to assess the effectiveness of passive vibration isolation [14,15].

The authors of this study conducted tests on the effectiveness of passive vibration isolation of selected viscoelastic materials in the FLD and Constrained Layer Damping (CLD) configuration at room temperature [16,17].

Due to the fact that passive vibration isolation is used to dampen vibrations of motor vehicles operating in various conditions, it is necessary to check the damping effectiveness of polymer materials in a wider temperature range.

The main scientific objective of this work is to determine the effect of temperature on the damping efficiency of viscoelastic materials (butyl rubber and bituminous material, FLD configuration), which are used in passive vibration isolation. The research results presented in this work are important both from a scientific and practical point of view and can be used in the automotive and aviation industries (in the process of designing passenger cabins with high acoustic comfort).

## 2. Materials and Methods

**Figure 1:**
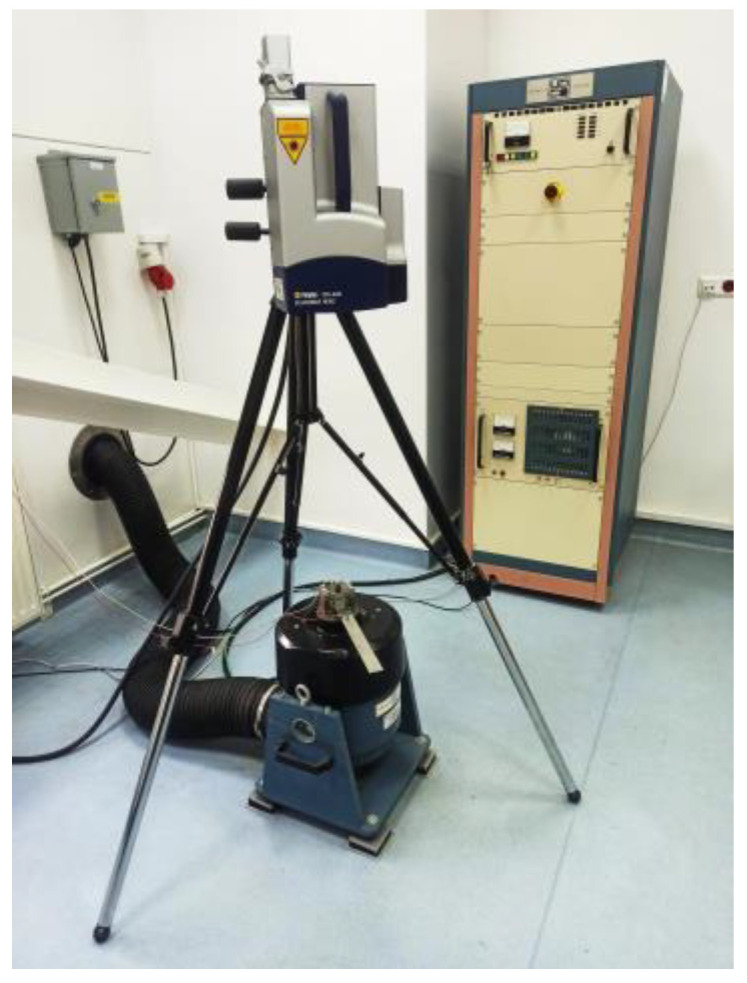
Electromagnetic vibration shaker and amplifier used in experimental modal analysis.

In order to determine the damping properties of tested viscoelastic materials, an experimental modal analysis was performed, using the Unholtz-Dickie UDCO-TA-250 electrodynamic vibration system (Unholtz-Dickie Corp., Wallingford, CT, USA) (Figure 1). The system was composed of a shaker, controller, amplifier, and the computer with software controlling the exciter operation.

**Figure 2:**
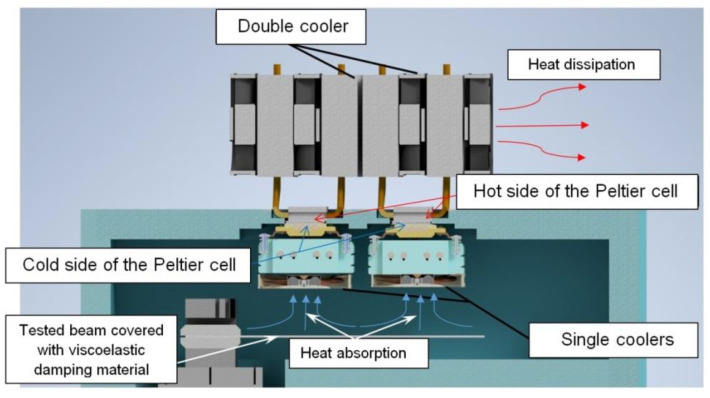
Diagram of the cooling chamber built above the vibration exciter.

In order to determine the influence of temperature on the effectiveness of passive vibration isolation, an isothermal cooling chamber was designed and constructed for the purposes of this work. This made it possible to place the tested beam with damping material in a tight chamber, inside which the temperature could be controlled. The Peltier cells were used to cool the isothermal chamber (Figure 2). The cooling chamber included a thermoelectric heat exchanger, a two-channel temperature controller (control output, 230 V—AC power supply), a 500 W power supply (max. voltage 12 V, current 41.7 A), a temperature sensor and a housing made of RAVATHERM board XPS (extruded polystyrene foam [18]) (Ravago Building Solutions Poland Sp. z o.o., Warsaw, Poland). This material has good thermal insulation properties. The housing took the form of a chamber attached to the exciter.

The principle of operation of the constructed thermoelectric heat exchanger was based on the properties of Peltier modules. When electric current flowed through the cell, heat was absorbed by one side of the module and released by the other. A double cooler was attached to one side of a single Peltier cell, and a single cooler to the other side (Figure 2). The contact surfaces of the cells and the radiators were covered with thermally conductive paste. Then, the entire structure was attached to the housing so that the double radiators (heat-releasing) were mounted outside and the single radiators (heat-absorbing) were mounted inside the cooling chamber. The coolers attached to the modules were intended to improve the heat exchange process.

**Figure 3:**
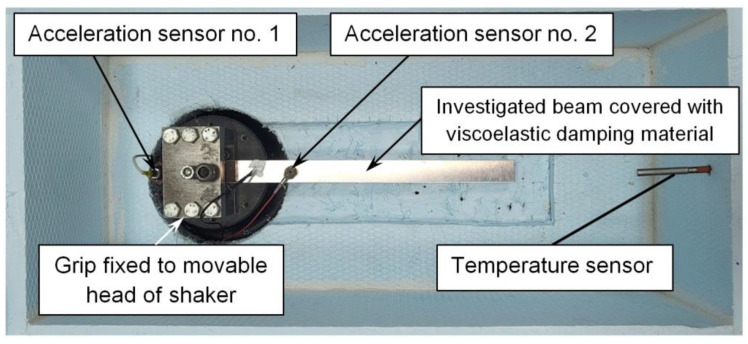
The interior of the cooling chamber with visible piezoelectric acceleration sensors, temperature sensor and the tested beam.

Figure 3 shows a view of the cooling chamber with the tested beam placed inside, attached to the movable vibrator head (the damping material is not visible because it is attached to the bottom of the beam).

**Figure 4:**
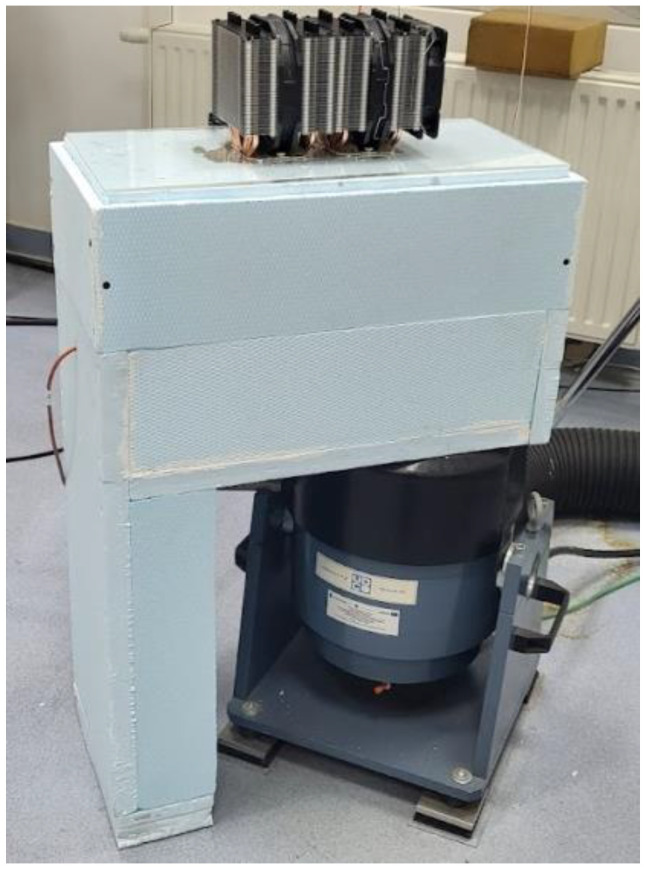
Vibration exciter with a closed cooling chamber and a heat sink for cooling the hot side of the Peltier cells.

Piezoelectric vibration sensors were used to determine the accelerations of the head and the tested object [19]. Sensor no. 2 was used to determine the acceleration (A_2_) of the beam. Sensor no. 2 was mounted at a distance of 50 mm from the beam restraint. Vibration sensor no. 1 was used to measure the acceleration of the exciter head (A_1_). The uncertainty estimate of acceleration measurement using the Endevco 256HX-100 piezoelectric sensor (Endevco Europe, Fribourg, Switzerland) (for 95% confidence) is ±1.7% (for the frequency range from 20 Hz to 120 Hz); ±1.7% (120 Hz–2500 Hz and ±2.7% (2500 Hz–4000 Hz). To maintain repeatability of measurements, the beam (at the beginning of the tests) was tightened to the exciter head with a constant torque using a torque wrench. During subsequent tests at lower temperatures, both the beam and the acceleration sensors were not dismantled, which ensured constant measurement conditions. Figure 4 shows the cooling chamber attached to the shaker.

**Figure 5:**
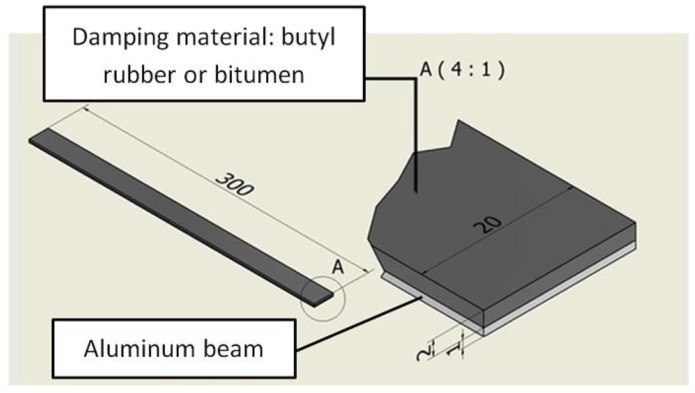
Dimensions and structure of specimen.

In the presented research, the Oberst beam method was used to determine the damping properties of the investigated materials [20,21]. The beam was made out of AW-2017A aluminum alloy [22,23]. The beam dimensions were as follows: 1 mm (thickness), 20 mm (width) and 300 mm (length) (Figure 5).

In the presented research, only the FLD configuration of the damping material was considered. The tested damping materials (butyl rubber and bitumen) with a self-adhesive layer were attached to the beam by pressing. In next step, the damping mats were rolled in order to obtain a high local pressure and in consequence a durable and strong connection. According to the manufacturers, mats made of butyl rubber and bituminous material do not contain toxic substances and are safe for humans (within the specified permissible operating temperatures). Mats used in passenger vehicles should also be flame retardant. The above-mentioned features are usually confirmed by appropriate certificates. The density of tested butyl rubber was 2 kg/dm^3^, while that of the bituminous material was 1.8 kg/dm^3^ [16]. The thickness of both damping materials (butyl rubber and bitumen) was 2 mm (Figure 5).

The storage modulus of damping materials depends on frequency and temperature. The storage modulus E′ of tested butyl rubber (in the frequency range 10–2500 Hz at 22 °C) reaches values from about 120 MPa to 230 MPa. At higher frequencies (2500–3700 Hz), the E’ modulus of butyl rubber increases from 230 MPa up to about 670 MPa. The storage modulus E’ of the tested bituminous material (at 22 °C) is within the range 55–600 MPa (in the frequency range from 10 Hz to 3000 Hz). The above values of the storage modulus were determined experimentally by the authors of this article through separate tests, which will be described in detail in a separate publication.

The samples were prepared according to Standard E756(05) (ASTM) [24]. The frequency range (40 Hz–4000 Hz) used in this research is within the range of excitation of piston and turbine engines (excitation from the rotor and crankshaft unbalance for the first and higher harmonics as well as excitation by propeller blades of turboprop aircraft). For the above reasons, the results obtained in this work can be used both in aviation and in the automotive industry. During the tests, the excitation frequency was changed linearly at a rate of 1 Hz/s. The vibration amplitude was measured using the ICP piezoelectric acceleration sensors on the shaker head (channel no 1) and on the beam (channel no 2). In the modal test, the intensity of acceleration of the shaker head was equal to 1 g (where 1 g = 9.81 m/s^2^). During the experiment, the frequency of excitation was increased.

## 3. Results and Discussion

The process of cooling the air inside the chamber was controlled by a temperature controller. During the experiment, modal analysis of two samples was performed (aluminum beam covered with a layer of butyl rubber and bituminous material) at temperatures of 22 °C, 18 °C, 15 °C, 10 °C, 5 °C and −2 °C. The last temperature value (−2 °C) was determined by the power of the designed cooling chamber. In modal analysis, the first mode of resonant vibrations was omitted because during this mode a large displacement amplitude of the beam occurred.

**Figure 6:**
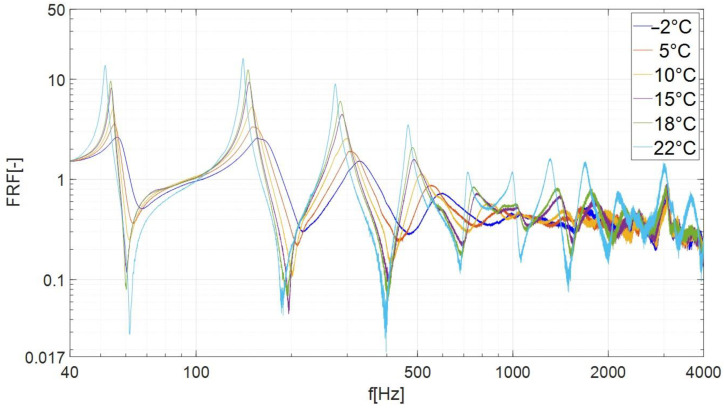
Frequency response curves of specimen damped by the butyl rubber in examined temperatures (frequency range of 40 Hz–4000 Hz).

**Figure 7:**
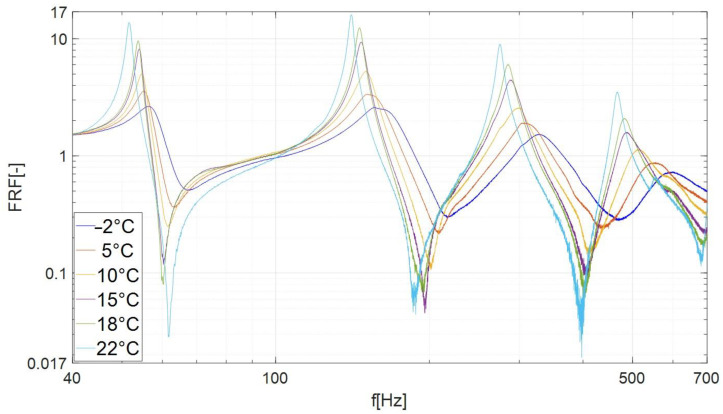
Frequency response curves of beam damped by the butyl rubber in examined temperatures (frequency range of 40 Hz–700 Hz, modes 2, 3, 4, 5).

In the performed tests, the LMS data-acquisition system recorded the beam response (vibration amplitude) with the resolution 0.1 Hz. As a main result of the experimental investigations, the amplitude–frequency plots were obtained for the considered samples. On the vertical axis of the obtained characteristics (Figure 6 and Figure 7), the frequency response function (FRF) was defined, which is used to determine the response of the system related to the magnitude of the excitation. The FRF values were calculated as follows: the signal from channel no. 2 (amplitude of acceleration A_2_ measured on the tested beam) was divided by the signal from channel no. 1 (amplitude of acceleration of the shaker head A_1_).

Figure 6 shows the FRF of a beam damped with the butyl rubber. The graph shows a number of peaks (local maxima), which we interpret as resonance vibrations of the specimen. The first peak, counting from the left, is identified with the second mode of vibration of the beam (the first mode of vibration was not tested due to the collision of the beam with the cooling chamber). The highest values of the FRF were recorded for the four lowest modes. For frequencies higher than 1 kHz, the FRF values of individual curves became similar; therefore, only modes 2, 3, 4 and 5 were subjected to further analysis (Figure 7).

The data obtained during the vibration analysis allowed the determination of modal parameters, which are presented in Table 1 and Table 2. Resonant frequencies were defined as f [Hz], and FRF amplitudes read in resonance were marked as A [-] and loss factors as η [-]. The symbol [-] in parameters A and η denotes a dimensionless value. In order to distinguish parameters measured at different temperatures, the temperature at which they were read is written in the subscript. For example, the parameter f_22°C_ (Table 1) corresponds to the resonance frequency of the beam tested at a temperature of 22 °C. The above-mentioned modal parameters were determined for the second, third, fourth and fifth modes of vibration and were summarized in the form of a table. To calculate the loss factor, the method proposed by the authors of [24] was used. The maximum values of the loss factor η (for each vibration mode, at different temperatures) indicate the highest damping intensity (the greatest energy dissipation).

The results presented in Figure 6 and Figure 7 and Table 1 and Table 2 indicate that when the temperature was lowered, the FRF amplitudes of subsequent modes decreased, and the corresponding resonance frequencies increased. The beam damped with butyl rubber showed the lowest energy dissipation (the weakest damping) at a temperature of 22 °C, where the lowest values of the loss factor η were recorded in the range of 0.027–0.033 (Table 1). As the temperature decreased, the attenuation increased and reached its maximum values at the lowest temperature tested (–2 °C, Table 2). For the above temperature, the loss factor (depending on the mode number) ranged from 0.152 to 0.289.

**Figure 8:**
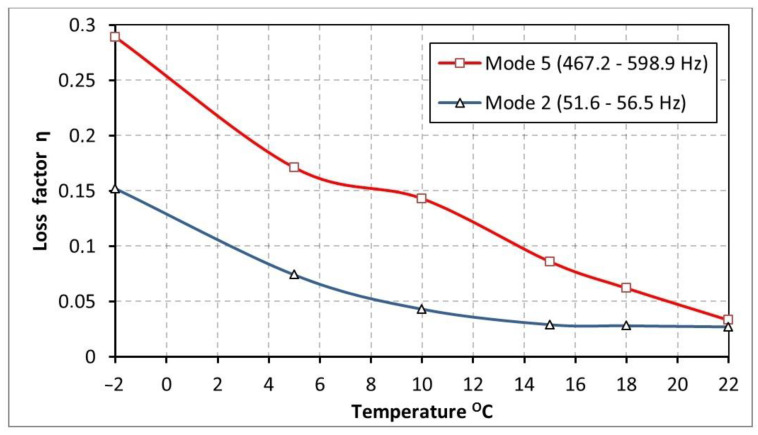
Loss factor η of beam damped by the butyl rubber as a function of temperature for selected modes (mode no. 2 (51.6 Hz–56.5 Hz) and mode no. 5 (467.2 Hz–598.9 Hz)).

The loss factor values for the two extreme modes (no. 2 and no. 5) visible in Figure 8 indicate that in the low frequency range (51 Hz–56 Hz), the damping properties of butyl rubber in the temperature range 15 °C–22 °C are almost the same (η = 0.027 ÷ 0.029). When lowering the temperature to −2 °C, the loss factor gradually increases to approximately 0.15. In the case of higher resonance frequencies (467.2 Hz–598.9 Hz), when the temperature is lowered (from 22 °C), a clear increase in damping intensity is observed (Figure 8). For this frequency range, the η at −2 °C reaches the highest value (0.289). The results presented in Figure 8 indicate that butyl rubber has the highest damping intensity at −2 °C, in the frequency range of about 470 Hz–600 Hz.

**Figure 9:**
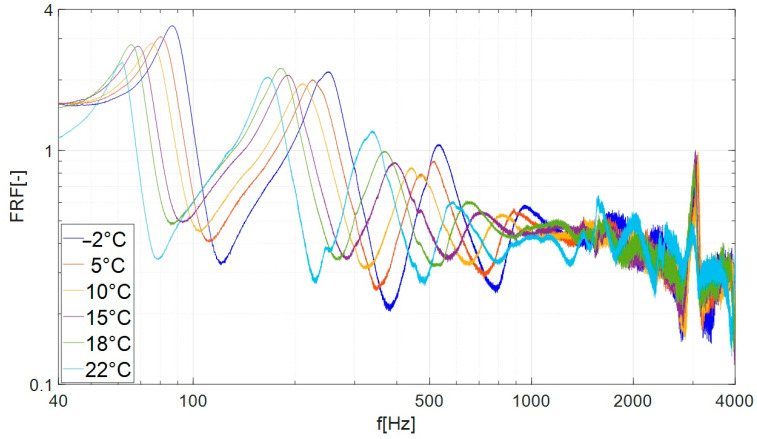
Frequency response curves of specimen damped by the bituminous material in the examined temperatures (frequency range of 40 Hz–4000 Hz).

**Figure 10:**
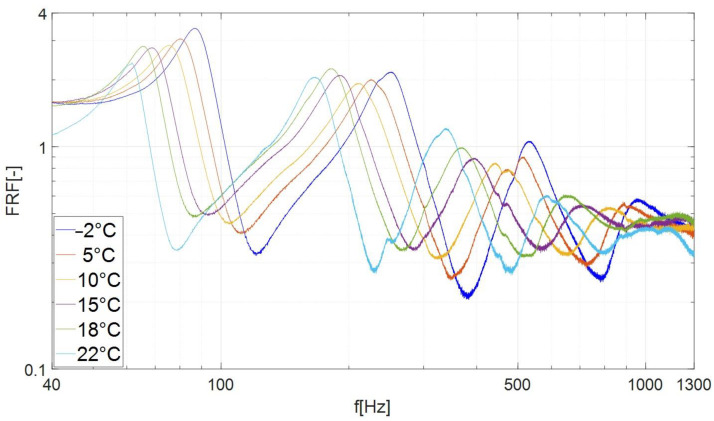
Frequency response curves of specimen damped by the bituminous material in the examined temperatures (frequency range of 40 Hz–1300 Hz, modes 2, 3, 4, 5).

Figure 9 shows the FRF values of a beam damped with bituminous material. On the vertical axis of the obtained characteristics (Figure 9 and Figure 10), the frequency response function (FRF) was defined, which is used to determine the response of the system related to the magnitude of the excitation. The test results indicate that lowering the temperature causes a significant increase in the frequency of resonance vibrations and a slight change in amplitude. For frequencies higher than 1 kHz, the FRF values of individual curves became so similar that only modes 2, 3, 4 and 5 were subjected to further analysis (Figure 10).

Figure 10 shows that in the case of mode no. 3, as the temperature decreases, the resonance characteristics of the beam damped with bituminous material shift to the right (towards higher frequencies) and the amplitude shows slight changes. A qualitative comparison of the characteristics presented in Figure 6, Figure 7, Figure 9 and Figure 10 indicates that the damping properties (in the context of changing the amplitude of resonance vibrations with lowering the temperature) of the bituminous material show significant differences compared to the properties of butyl rubber.

In order to quantitatively compare the modal parameters of the bituminous material, Table 3 and Table 4 present the values of resonant frequency, amplitude of FRF during resonance and loss factor η for the temperature range from −2 °C to 22 °C.

The test results (Table 3 and Table 4) indicate that lowering the temperature of the bituminous material resulted in a significant increase in the resonance frequencies of the beam. In the case of mode no. 2, as the temperature decreased, the FRF amplitude increased. For modes no. 3, 4 and 5, no trend in amplitude change was observed (irregular oscillations occurred). The best vibration damping was achieved at a temperature of 10 °C for each of the tested modes.

**Figure 11:**
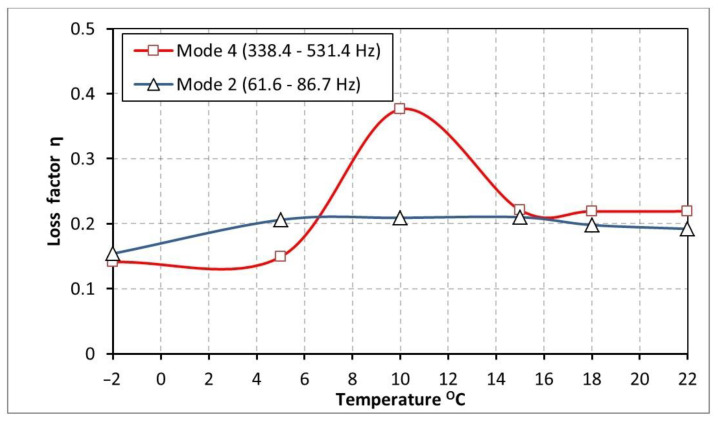
Loss factor η of beam damped by bituminous material as a function of temperature for selected modes (mode no. 2 (61.6 Hz–86.7 Hz) and mode no. 4 (338.4 Hz–531.4 Hz)).

In order to quantitatively compare the damping intensity of a beam with bituminous material, Figure 11 shows the course of the loss factor values as a function of temperature for mode 2 (frequency range 61.6 Hz–86.7 Hz) and mode 4 (frequency range 338.4 Hz–531.4 Hz). When selecting mode no. 4 (shown in Figure 11, as the best one for comparison purposes), a frequency criterion was used (the highest degree of frequency coverage with the mode no. 5 for the butyl rubber (Table 1 and Table 2).

The graphs presented in Figure 11 indicate that the temperature (in the analyzed range from −2 °C to 22 °C) does not significantly affect the damping properties of the bituminous material for mode 2 (frequency range 61.6 Hz–86.7 Hz) where the loss factor values oscillates around 0.2. In the case of mode no. 4 (338.4 Hz–531.4 Hz), there is a high local increase in damping efficiency visible at 10 °C (η = 0.376).

## 4. Conclusions

In this paper, the influence of temperature on the damping properties of the butyl rubber and the bituminous material was investigated. In first part of the work, the steps of designing and operating the cooling chamber were described. Moreover, the main components of the vibration system, measuring and important control parameters were explained. Next, an experimental modal analysis of a beam covered with the tested viscoelastic damping materials (in the FLD configuration) was performed. As a main result of the performed investigation, FRFs were obtained for the tested specimens. On the base of FRF analysis, the frequencies and amplitudes of resonant vibrations were obtained. In order to assess the damping efficiency, the loss factor of samples damped with both the butyl rubber and the bituminous material was determined in the temperature range of −2 °C to 22 °C, for different modes of vibration (different frequencies).

As a result of the investigations performed, the following conclusions were formulated:

1. The comparison of the loss factor value of a beam damped by the butyl rubber (mode 2, frequency range of 51–56 Hz, Table 1 and Table 2, Figure 8) with the loss factor value of a beam covered by bitumen (mode no. 2, frequency range 61.6 Hz–86.7 Hz, Table 3 and Table 4, Figure 11) shows that in the low frequency range, the bituminous material has the highest damping efficiency, for all of the tested temperatures.
2. The results of the experimental tests showed that in the higher frequency range (mode no. 5, 467.2–598.9 Hz, Table 1 and Table 2, Figure 8 and mode no. 4, 338.4 Hz–531.4 Hz, Table 3 and Table 4, Figure 11), the bitumen has better damping properties in the temperature range of 6–22 °C than the butyl rubber. For lower temperatures (from −2 °C to 6 °C), the butyl rubber dampens vibrations better than the bituminous material.
3. The damping properties of butyl rubber depend strongly on temperature. The loss factor of the beam damped with butyl rubber at a temperature of 22 °C is the lowest (0.027–0.033). Lowering the temperature of butyl rubber causes multiple increases in the loss factor value of 0.152 (mode no. 2) and 0.289 (mode no. 5).
4. In the range of low resonant frequencies (mode no. 2, 61.6 Hz–86.7 Hz), the loss factor values of bitumen oscillated around the value of 0.2. For the above-mentioned frequency range, the temperature does not significantly affect the damping properties of the bituminous material.
5. In the higher frequency range (mode no. 4, 338.4 Hz–531.4 Hz), the damping properties of the bituminous material depend on the temperature. For a temperature of 10 °C, a local maximum of the loss factor value n = 0.376 can be observed (the highest vibration damping ability). In the temperature range of −2 to 5 °C, the attenuation is about 3 times smaller (n = 0.15), while for temperatures of 15–22 °C, the loss factor reaches values of about 0.2.

The results obtained in this study show that bituminous material and butyl rubber have different vibration damping properties. These properties depend, among other things, on the frequency of vibrations and on the operating temperature. Thus, in order to design passive vibration isolation well, it is necessary to conduct a thorough analysis of the operation of the structure in terms of vibration frequency and operating temperature.

The work of a pilot or vehicle driver in a noisy environment affects his mental condition and increases fatigue. As a result of this phenomenon, the reaction time increases and the operator’s ability to make the right decision decreases. For the above reasons, scientific work that may result in improved acoustic comfort in the cabin of an airplane or car has an indirect impact on increasing transport safety. The research results presented in this work are not only of scientific but also practical importance and can be used in the design of motor vehicles, rail vehicles and aircraft structures.

The temperature range at which the damping effectiveness of bituminous material and butyl rubber was tested (from −2 °C to 22 °C) was determined by the limited cooling power of the chamber equipped with Peltier cells. The given temperature range partially coincides with the operating temperature of aircraft and motor vehicles in a moderate climate. As part of further research, the authors plan to expand the temperature range from about −40 °C to +50 °C, which coincides with most operating temperatures in the aviation and automotive (in different climatic zones) fields. Additionally, it is also planned to investigate other alternative damping materials (e.g., polyurethane foam, rubber, silicone).

## Figures and Tables

**Table 1:** Frequency of resonant vibration, amplitude of FRF during resonance and loss factor η for beam damped by the butyl rubber in temperature 22 °C, 18 °C and 15 °C.

Temp	22 °C				18 °C		15 °C		
No. of Mode	$f_{22{}^{\circ}C}$ [Hz]	$A_{22{}^{\circ}C}$ [-]	$\eta_{22{}^{\circ}C}$ [-]	$f_{18{}^{\circ}C}$ [Hz]	$A_{18{}^{\circ}C}$ [-]	$\eta_{18{}^{\circ}C}$ [-]	$f_{22{}^{\circ}C}$ [Hz]	$A_{22{}^{\circ}C}$ [-]	$\eta_{22{}^{\circ}C}$ [-]
**2**	51.6	13.8	0.027	53.8	9.6	0.028	54.1	8.2	0.029
**3**	140.7	16.1	0.024	145.8	12.4	0.032	147.2	9.4	0.043
**4**	275.1	8.9	0.029	285.6	6	0.051	288.6	4.5	0.064
**5**	467.2	3.5	0.033	482.3	2.1	0.062	486.2	1.6	0.086

**Table 2:** Frequency of resonant vibration, amplitude of FRF during resonance and loss factor η for beam damped by the butyl rubber in temperatures of 10 °C, 5 °C and −2 °C.

Temp	10 °C			5 °C			−2 °C		
No. of Mode	$f_{10{}^{\circ}C}$ [Hz]	$A_{10{}^{\circ}C}$ [-]	$\eta_{10{}^{\circ}C}$ [-]	$f_{5{}^{\circ}C}$ [Hz]	$A_{5{}^{\circ}C}$ [-]	$\eta_{5{}^{\circ}C}$ [-]	$f_{-2{}^{\circ}C}$ [Hz]	$A_{-2{}^{\circ}C}$ [-]	$\eta_{-2{}^{\circ}C}$ [-]
**2**	54.7	5	0.043	55.3	3.6	0.074	56.5	2.6	0.152
**3**	150.2	5.3	0.076	151.1	3.4	0.161	156.1	2.6	0.228
**4**	299.8	2.6	0.107	308.9	1.9	0.167	328.9	1.5	0.206
**5**	513.6	1.1	0.143	554.4	0.9	0.171	598.9	0.7	0.289

**Table 3:** Frequency of resonant vibration, amplitude of FRF during resonance and loss factor η for beam damped by the bituminous material in temperatures of 22 °C, 18 °C and 15 °C.

Temp	22 °C				18 °C		15 °C		
No. of Mode	$f_{22{}^{\circ}C}$ [Hz]	$A_{22{}^{\circ}C}$ [-]	$\eta_{22{}^{\circ}C}$ [-]	$f_{18{}^{\circ}C}$ [Hz]	$A_{18{}^{\circ}C}$ [-]	$\eta_{18{}^{\circ}C}$ [-]	$f_{22{}^{\circ}C}$ [Hz]	$A_{22{}^{\circ}C}$ [-]	$\eta_{22{}^{\circ}C}$ [-]
**2**	61.6	2.4	0.154	65.4	2.8	0.206	68.9	2.8	0.209
**3**	166.6	2.1	0.182	181.8	2.2	0.231	190.1	2.1	0.221
**4**	338.4	1.2	0.229	367.9	1	0.219	396.1	0.9	0.221
**5**	584.1	0.6	0.287	655.6	0.61	0.31	704.1	0.6	0.217

**Table 4:** Frequency of resonant vibration, amplitude of FRF during resonance and loss factor η for beam damped by the bituminous material in temperatures of 10 °C, 5 °C and −2 °C.

**Temp**	10 °C				5 °C		−2 °C		
**No.** **of** **Mode**	$f_{10{}^{\circ}C}$ **[Hz]**	$A_{10{}^{\circ}C}$ **[-]**	$\eta_{10{}^{\circ}C}$ **[-]**	$f_{5{}^{\circ}C}$ **[Hz]**	** $A_{5{}^{\circ}C}$ ** **[-]**	$\eta_{5{}^{\circ}C}$ **[-]**	$f_{-2{}^{\circ}C}$ **[Hz]**	$A_{-2{}^{\circ}C}$ **[-]**	$\eta_{-2{}^{\circ}C}$ **[-]**
**2**	75.8	2.9	0.21	80.1	3.1	0.198	86.7	3.4	0.192
**3**	210.3	1.9	0.253	225.7	2	0.238	251.1	2.2	0.212
**4**	441.9	0.8	0.376	515.5	0,9	0.149	531.4	1.1	0.141
**5**	815.7	0.5	0.711	889.5	0.6	0.195	950.4	0.6	0.302

## Data Availability

The original contributions presented in the study are included in the article, further inquiries can be directed to the corresponding author.
